# Assessment of irreducible aspects in developmental hip dysplasia by magnetic resonance imaging

**DOI:** 10.1186/s12887-020-02420-2

**Published:** 2020-12-05

**Authors:** Huihui Jia, Liang Wang, Yan Chang, Yongrui Song, Yuqi Liu, Fuyong Zhang, Jie Feng, Xiaodong Yang, Mao Sheng

**Affiliations:** 1 Department of Radiology, Children’s Hospital of Soochow University, Suzhou, 215000 PR China; 2 Department of Orthopaedics, The Affiliated Suzhou Science & Technology Town Hospital of Nanjing Medical University, Suzhou, 215000 PR China; 3 Suzhou Institute of Biomedical Engineering and Technology, Chinese Academy of Sciences, Suzhou, 215163 PR China; 4 Department of Orthopaedics, Children’s Hospital of Soochow University, Suzhou, 215000 PR China

**Keywords:** Irreducible factors, Developmental dysplasia of the hip, MR imaging

## Abstract

**Background:**

The developmental dysplasia of the hip (DDH) can cause a wide range of pathological changes, and often requires surgical treatment. Preoperative evaluation is very important for DDH. We aimed to assess the diagnostic capability of magnetic resonance imaging (MRI) for irreducible aspects preventing hip reduction in DDH.

**Methods:**

A total of 39 pediatric patients who received DDH evaluation in pediatric orthopedics from January 2015 to December 2019 were included. The samples included 4 cases of bilateral DDH and 35 cases of unilateral DDH, a total of 43 hip joint samples. All patients underwent surgical treatment, pathological examination and MRI of hip joint.

**Results:**

With pathological results or intraoperative findings as the gold standard, the sensitivity and specificity of MRI were 90.3% and 83.3% for the affected labrum, 92% and 83.3% for thickening of the round ligament, 90.0% and 91.3% for atrophy of the iliopsoas muscle, and 100% and 100% for fibrofatty pulvinar tissue and joint effusion, respectively.

**Conclutions:**

The MRI showed an extraordinary capability of detecting these irreducible factors and helped surgeon choose the appropriate treatment strategies.

## Background

Developmental dysplasia of the hip (DDH) encompasses a wide spectrum of pathology, ranging from complete fixed dislocation at birth to asymptomatic acetabular dysplasia in adulthood [1], which can negatively affect hip development and can lead to isolated acetabular dysplasia, subluxation, complete dislocation of the hip joint, or permanent abnormal gait [2, 3]. The children with untreated persistent DDH may suffer from pain due to a series of anatomical changes such as increased abnormal articular pressure or increased tension on a smaller contact area during the process of growth and development. With the development of DDH, the degeneration of the articular cartilage, early coxarthrosis, and ischemic necrosis of the femoral head would likely happen in children [4].

Result from less invasive and more effective, early diagnosis and treatment plays important roles in restoring the normal relationship between the acetabulum and femoral head and avoiding further complications that come with growth [5]. According to the American College of Radiology (ACR) guides, the most important diagnosis method of DDH is on the basis of the results of physical examination using the Ortolani’s sign and Barlow’s test, though in 1977, Jones [6] reported that preliminary physical examination did not satisfy the clinical needs of DDH diagnosis due to low sensitivity. Several imaging techniques were also emerged in clinical, including ultrasonography (US), digital radiography (DR), and magnetic resonance imaging (MRI), which improved the diagnostic sensitivity of DDH. US is the preferred imaging modality for evaluating infants younger than 4 months [7]. However, US is not so reliable in assessing the depth of femoral head coverage due to the measurements of acetabular angles depend on the image readers and are highly variable, and limited in identifying intrinsic obstacles to reduction such as the hypertrophied pulvinar after capital femoral epiphyseal ossification. Therefore, high rates of late diagnosis in DDH persist in the context of selective ultrasound screening [8, 9]. DR was commonly applied for diagnosing DDH in patients older than 6 months of age, and the cons is it’s not helpful in identifying secondary intra-articular changes and is associated with the inevitable risk of radiation [10]. Moreover, both US and DR are difficult to diagnose preoperatively inverted labrum, hypertrophied ligamentum teres, and shortened iliopsoas muscle.

With the rapid development of imaging technologies, MRI has been shown to be advantageous in the detection of bony structures as well as cartilaginous and soft-tissue structures. Although there are significant individual differences in the location and degree of acetabular dysplasia [11], MRI can provide superior soft tissue resolution in cross-sectional imaging profiles without ionizing radiation and has been advocated for the assessment of acetabular morphology and growth disturbance or deficiencies of the capital femoral epiphysis, the discrimination of ossified and unossified components, especially the identification of joint congruity and obstacles to prevent the reduction of hip dislocation, and the detection of unexpected complications after surgical reduction [12]. Benefit from MRI, it’s convenient for clinicians, regardless of experience level, subspecialty, or geographic origin, to assess the quality of hip reduction and predict surgical reduction of avascular necrosis (AVN). Magnetic resonance imaging provides a more reliable interpretation than standard X-rays and reduces radiation exposure [13, 14].

However, there is few studies to systematically explore the abilities of MRI in DDH to detect the various obstacles, including thickened fibrofatty pulvinar tissue, joint effusion, thickened ligamentum teres, inversion of the labrum, hypertrophy of the cartilage of the acetabular roof, and iliopsoas muscle atrophy, to concentric reductions, which is the treatment goal of DDH and critical for better prognosis [15, 16].

The present study aimed to evaluate the capability of MRI for precisely detecting the irreducible mechanisms preventing concentric hip reduction in DDH before surgery, and its applicability in guiding clinical treatment.

## Methods

### Subjects

From January 2015 to May 2019, 168 children with DDH were treated at Children’s Hospital, Suzhou, China. 44 of them received the open reductions and related MR images were collected.

Three patients, who had been previously treated in other hospitals were excluded from this study, as well as one due to poor quality of MR images and another one with teratologic dislocations. A total of 39 primary patients (9 boys and 30 girls) were included in this study with a mean age of 26.4 ± 5.3 months (range, 18–48 months). Among these patients, 4 had bilateral DDH and 35 had unilateral involvement, giving a total of 43 affected hips. Although the severities of these patients were different, the imaging and pathological features are similar. Informed consents were obtained from the parents of each patient. Ethics committee approval was obtained from the Institutional Review Board of Children’s Hospital of Soochow University (Suzhou, China), and was performed in accordance with the ethical guidelines of the Declaration of Helsinki (Ethical batch number:2015KS006).

### Inclusion criteria

① Patients with DDH were diagnosed clinically, and MRI scan was performed before the operation; ② no abnormal findings were found in physical examination of neuromuscular system; ③ no previous treatment was performed; ④ the operation was open reduction of hip joint. The above four points need to be met at the same time.

### Exclusion criteria

①Patients with DHH who have received non-surgical or surgical treatment (secondary dislocation of hip joint); ② patients with history of hip infection or suppurative hip arthritis in neonates or infants; ③ patients with other diseases; ④ patients with nonstandard clinical data, MRI, operation records or incomplete results. The case would be excluded when any of the exclusion criteria was satisfied. 

### MRI data acquisition

The MRI examinations were performed before the open reduction on a 3.0T scanner (Siemens AG, Magnet, Germany) using a baby array coil placed anterior and posterior to the hips. All children were sedated before the MR examination. The children were then positioned supine inside the scanner with both legs in a symmetrical neutral position. Then, localizer T1-weighted (T1-W) spin-echo (SE) images (repetition time [TR], 796 ms; echo time [TE], 9.43 ms; time of acquisition, 3 min 55 s) were obtained in the coronal and axial planes. The coronal sequence in T2-weighted fast-spin-echo (TR/TE, 4000/103 ms; time of acquisition, 2 min 40 s) and short inversion time inversion recovery (STIR; TR/TE, 5400/42 ms; time of acquisition, 2 min 20 s) were obtained. Slice thickness was 3.0 mm for all the sequences, and the interslice gap was 1.0 mm. The field of view was 300 × 300 mm^2^ and matrix was 256 × 256 for all images [17].

### MRI image analysis

All the MR images were uploaded to the Picture Archiving and Communication Systems (PACS; Neusoft, Shenyang, China) and sent to the AW 4.6 workstation (GE Healthcare, Waukesha, WI, USA). The films were read independently by two radiologists who were with the same seniority and both unknown about the subjects’ clinical diagnosis and progress as the attending physician. Pathological changes such as fibrofatty pulvinar tissue, joint effusion, thickening of ligamentum teres, inversion of labrum, hypertrophy of the cartilage of the acetabular proof and atrophy of the iliopsoas muscle were analyzed on the AW4.6 workstation. According to the corresponding image manifestations and applying the pathological examination results or intraoperative findings as the gold standards, the sensitivities, specificities, positive predictive values (PPV), negative predictive values (NPV), and accuracies to assess the diagnostic capabilities of MRI for the pathological changes were obtained. The width of the iliopsoas muscle at the level of femoral neck was used as a criterion for muscle atrophy.

### Diagnostic criteria

The severity of DDH was classified into grade I, II and III by observing the location of the femoral head on the coronal plane of MRI according to Dunn’s criteria [18]. In grade I, only the femoral head moves outward, and the center is still at the level of acetabulum; in grade II, the femoral head moves outward, but the center is beyond the upper edge of acetabulum; in grade III, the femoral head completely dislocates from the acetabulum.

### Statistical analysis

Statistical analysis was performed using *SPSS 22* package (*SPSS*, Chicago, Illinois). A *Kappa* test was used to assess the consistency of MRI manifestations with the results of the pathological analysis or intraoperative findings. The interobserver variability was assessed by intra-class correlation coefficient (ICC). For results of both the ICC and the *Kappa* test, a value of > 0.75 was regarded as excellent, a value of 0.40–0.75 was regarded as fair to good, while a value < 0.40 was poor.

## Results

**Fig. 1:**
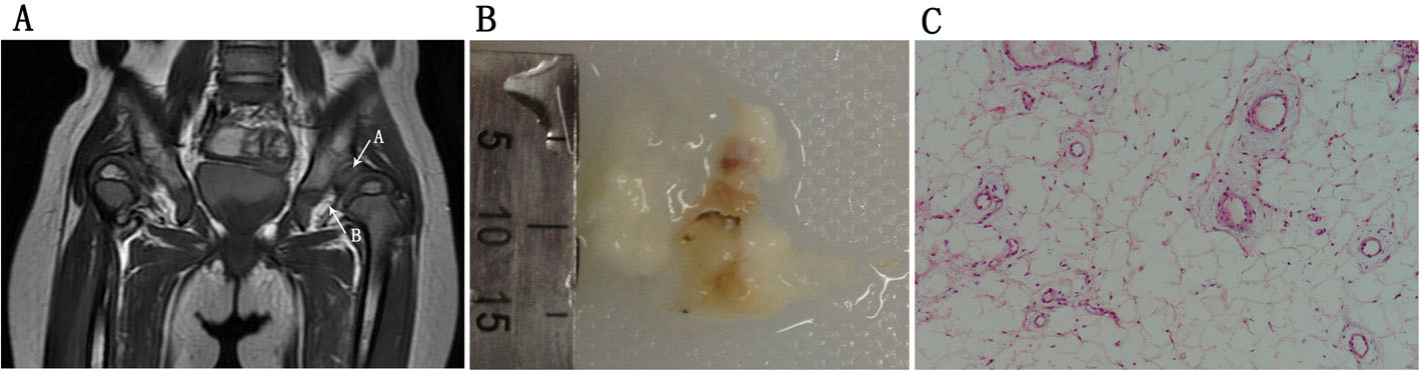
**a** T1WI: Case of a 2-year-old girl with grade III DDH on coronal slices shows inversion of the labrum (*arrow A*) and fibrofatty pulvinar tissue (*arrow B*). **b** Gross specimen:Gross specimen of the fibrofatty pulvinar tissue shows light yellow appearance. **c** Pathology: Light microscopy reveals hyperplasia of fat cells. (hematoxylin and eosin stain; magnification ×**4**00)

**Fig. 2:**
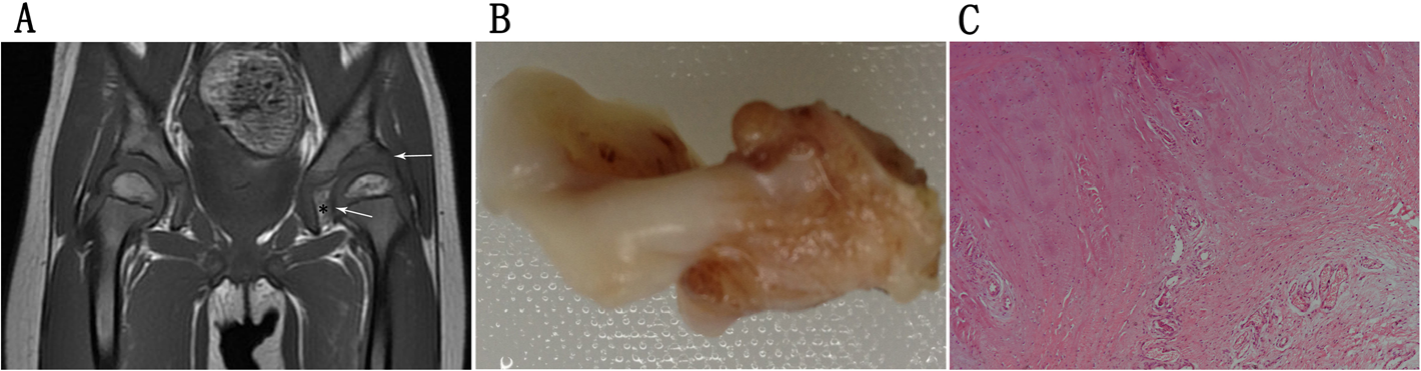
**a** T1WI:Case of a 19-month-old girl with grade II DDH on coronal slices shows inversion of the labrum (*horizontal arrow*), fibrofatty pulvinar tissue (*asterisk*), and thickening of the round ligament (*oblique upward arrow*). **b** Gross specimen: Gross specimen of thickening of the round ligament removed from the left hip measuring 30mm. **c** Pathology:Light microscopy reveals dense fibrous tissue hyperplasia with focused hyaline degeneration. (hematoxylin and eosin stain; magnification ×200)

**Fig. 3:**
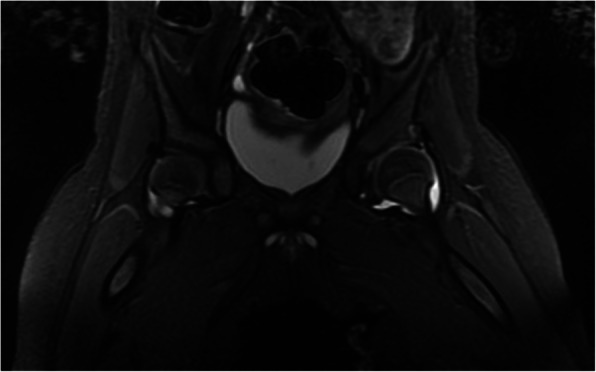
T2WI STIR: Case of a 7-month-old girl with joint effusion on STIR coronal section images shows a high-intensity signal on the left hip

**Fig. 4:**
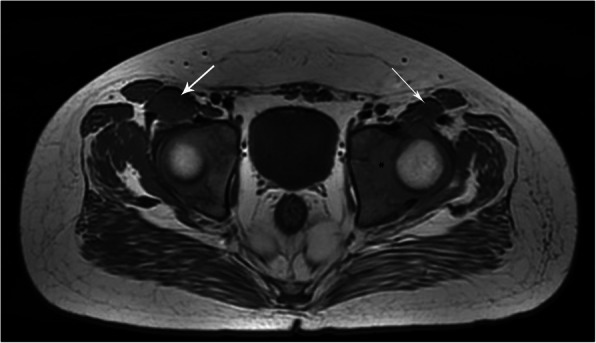
T1WI: Case of a 7-month-old girl with atrophy of the left iliopsoas muscle (*arrow*) and the right normal muscle (*arrow*) shows an intermediate-intensity signal on T1-weighted axial section images. The left iliopsoas muscle width decreases

**Fig. 5:**
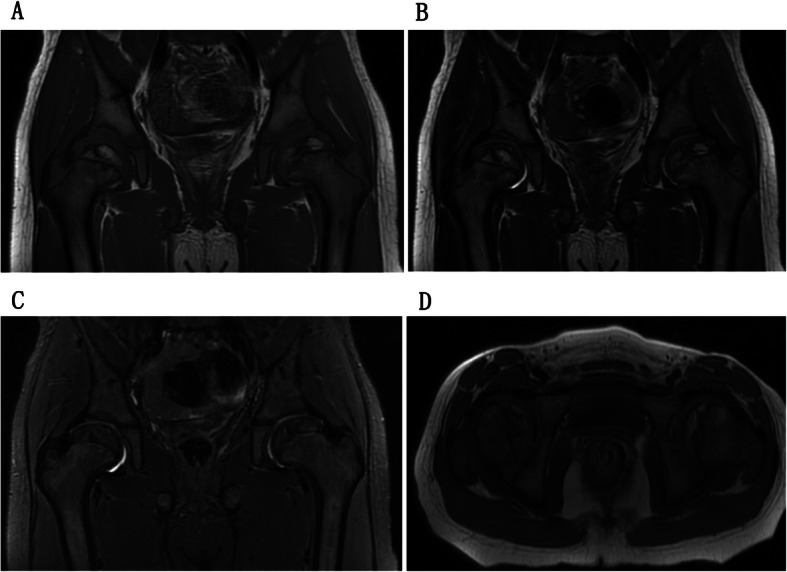
Case of a 6-year-old girl with bilateral avascular necrosis of femoral head shows heterogeneous signal multiple bone fragments on the coronal T1WI (**a**), T2WI (**b**), STIR (**c**) and transverse T1WI (**d**)**.**

Forty-three of the affected hips received open reductions after diagnosis. As a result, 31 inversed labrum, 31 fibrofatty pulvinar tissue, 25 thickened round ligament, 26 joint effusion, and 20 iliopsoas muscle atrophy were confirmed in the surgeries. It was showed that thickened fibrofatty pulvinar tissue appeared in 31 hips with intensified signal on T1-W coronal slices (Fig. 1). Ligamentum teres was voluminous in 26 hips, which produced low-intensity signals in all sequences (Fig. 2). Joint effusion was found in 26 hips with intensified signals on STIR coronal section images (Fig. 3). The labrum was inversed in the joint and intermediate-intensity signal was obtained on T1-W sequences (Fig. 1). Iliopsoas muscle atrophy appeared in 20 hips, which produced intermediate-intensity signals on T1-W axial section images and showed decreased muscle width compared with the other side (Fig. 4). Four patients with thickened round ligament were followed up for 3.5 years after DDH surgery, 2 of them developed bilateral AVN of femoral head. It’s found that femoral head had heterogenous signal intensity and non-integrality osseous morphology in MRI images (Fig. 5).

With pathological results and intraoperative findings as the gold standards, the sensitivity, specificity, PPV, NPV and accuracy for detecting affected labrum by MRI were 90.3%, 83.3%, 93.3%, 76.9%, and 88.4%, respectively (*kappa* = 0.718, 95% confidence interval (CI) (0.488,0.948); Table 1), 92.0%, 83.3%, 88.5%, 88.2%, and 88.4% for detecting thickened round ligament respectively (*kappa* = 0.759, 95% CI (0.561, 0.957); Table 1) and 90.0%, 91.3%, 90.0%, 91.3%, and 90.7% for iliopsoas muscle atrophy respectively (*kappa* = 0.813, 95% CI (0.639, 0.988); Table 1). The corresponding values for both fibrofatty pulvinar tissue and joint effusion were 100%, 100%, 100% and 100%.

**Table 1:** The detection of labrum, ligamentum teres and the contracture of the iliopsoas muscle by MRI and pathology

	Pathology								
MRI	Labrum			Ligamentum teres			Contracture of the iliopsoas muscle		
	(+)	(—)	Total	(+)	(—)	Total	(+)	(—)	Total
(+)	28	2	30	23	3	26	18	2	20
(—)	3	10	13	2	15	17	2	21	23
Total	31	12	43	25	18	43	20	23	43

Count data were assessed by the Kappa test. The consistency of MRI manifestations with the pathological results for labrum, Kappa = 0.718, 95% CI (0.488, 0.948); for ligamentum teres, Kappa = 0.759, 95% CI (0.561, 0.957); for contracture of the iliopsoas muscle, Kappa = 0.813, 95% CI (0.639, 0.988)

The Kappa values indicate fairly good consistencies between MRI imaging and the pathology

Global interobserver consistencies between the two senior radiologists of MRI were as follows (Table 2): for fibrofatty pulvinar tissue and joint effusion (ICC = 1.00), for thickening of the ligamentum teres (ICC = 0.72, 95% CI (0.490, 0.850)), for inversion of the labrum (ICC = 0.88, 95% CI (0.777, 0.934)) and for atrophy of the iliopsoas muscle (ICC = 0.93, 95% CI (0.870, 0.962)), indicating excellent agreement.

**Table 2:** Global interobserver consistency between the two senior radiologists of MRI

	Doctor 1														
Doctor 2	Labrum			Ligamentum teres			Contracture of the iliopsoas muscle			Fibrofatty			Joint Effusion		
	(+)	(—)	Total	(+)	(—)	Total	(+)	(—)	Total	(+)	(—)	Total	(+)	(—)	Total
(+)	23	0	23	24	0	24	18	0	18	31	0	31	26	0	26
(—)	5	15	20	10	9	19	3	22	25	0	12	12	0	17	17
Total	28	15	43	34	9	43	21	22	43	31	12	43	26	17	43

The interobserver variability was assessed by intra-class correlation coefficient (ICC). Global interobserver consistencies between the two radiologists for labrum, ICC = 0.88, 95% CI (0.777, 0.934); for ligamentum teres, ICC= 0.72, 95% CI (0.490, 0.850); for contracture of the iliopsoas muscle, ICC = 0.93, 95% CI (0.870, 0.962); for fibrofatty pulvinar tissue and joint effusion, ICC = 1.00

The ICC values indicate fairly good consistencies between the two senior radiologists of MRI

## Discussion

Despite the cost and possible requirement of additional sedatives, the advantages of MR imaging are considerable in DDH assessment, including the absence of radiation, possibility of imaging in multiple planes, and higher resolution and contrast between bony and cartilaginous components [19]. The high accuracy of MRI in demonstrating the details of DDH has been described in previous literature by comparing with anatomical preparations in cadavers [20]. More recently, MRI is being used in the diagnosis of the anatomical obstructions to reduction, such as fibrofatty pulvinar tissue, joint effusion, thickened ligamentum teres, inversed and thickened labrum, and iliopsoas muscle atrophy. Most of these studies evaluated one of these characteristics and confirmed the role of MRI in the diagnosis of DDH [20–22]. In these papers, the diagnostic values of MRI are evaluated comprehensively. And the diagnostic capability of MRI was in good agreement with pathological examination or surgical findings.

In the evaluation of 43 affected hips, the fibrofatty pulvinar tissue and joint effusion were easily detected by MRI due to different signal manifestations in T1WI, T2WI and STIR fat suppression sequences for tissues with fat or liquid. T1WI and T2WI showed high signal for fat and liquid signals, while stir fat suppression sequence showed low signal intensity. T1WI and Flair showed low signal for liquid signals, while T2W showed high signal. MRI can show fat and liquid sensitively, and the coincidence rate with pathological diagnosis is 100%. However, Benjamin et al. [23] reported that a small amount of adipose tissue and joint effusion can be completely absorbed once the femoral head is relocated and well covered by the acetabulum.

The labrum is one of the most important structures of the hip joint, which can improve the lateral coverage of the femoral head and deepen the joint. The labrum is typically everted with mild joint dislocation, but it may invaginate into the hip joint together with a capsular fold and inhibit reduction in the case of complete joint dislocation. In order to achieve a concentric reduction, the everted, inverted, and hypertrophied labrum require radial cuts [17]. In this study, the sensitivity and specificity of detection of the affected labrum were 90.3% and 83.3%, respectively, which were consistent with previous reports [24–26].

As another secondary adaptive change, thickened or elongated ligamentum teres often requires surgical excision, especially in grade III DDH. The stress is concentrated on a small area of the acetabular roof and may lead to high rates of avascular necrosis of the femoral head. Therefore, the hypertrophied ligament teres should be removed in the surgery to achieve optimal results. Devitt et al. [27] reported that MRI can be used to rule out partial tears of the ligamentum teres with sufficient sensitivity (91%) and PPV(67%) and accuracy (64%) but relative low specificity (9%) and NPV (31%). In our study, 25 hips with thickened ligamentum teres were identified in the surgery, 23 of them were detectable by MRI. And the sensitivity (92%) and specificity (83.3%), PPV (88.5%), NPV (88.2%) and accuracy (88.4%) for detecting thickened ligamentum teres were much higher in our reports, which may be partly attributed to higher resolution 3.0T MRI used in this study. AVN is one of the most common serious complication of DDH [28]. In the study, bilateral AVN were shown on 2 of 4 patients with elongated ligamentum teres during 3.5 years follow up, which led to the surgery. One of the possible reasons is that ligamentum teres exerts abnormally high pressure on ossific nucleus for a long time before surgery, indicating the early diagnosis by MRI and surgical removal of elongated ligamentum teres will be the key to decrease the occurrence of AVN.

The iliopsoas tendon passes between the acetabulum and the displaced femoral head and may obstruct concentric reduction [29]. The iliopsoas muscle atrophy often occurs on the affected hip as a result of biomechanical changes after DDH. Therefore, complete tenotomy of the iliopsoas muscle is recommended due to its advantages such as lower risk of avascular necrosis of the femoral head and decreased pressure on the hip joint. Keeney et al. [24] reported that the three-dimensional (3D) MRI was quite useful in diagnosing the iliopsoas muscle atrophy and was helpful in predicting the reduction difficulty. In our study, preoperative MRI has a high diagnostic efficiency, and its sensitivity, specificity, positive predictive value, negative predictive value and accuracy are highly consistent with postoperative pathology. The preoperative MRI would be helpful to identify the iliopsoas muscle atrophy.

In our research, there was good consistency between the two senior radiologists of MRI in thickening of the ligamentum teres, inversion of the labrum, atrophy of the iliopsoas muscle, fibrofatty pulvinar tissue and joint effusion (ICC > 0.72), indicating that MRI has good repeatability in the diagnosis of DDH and is suitable for preoperative diagnosis and postoperative evaluation and follow-up.

There were significant differences in the number of boys and girls in the study, with European scholars reporting a male-to-female ratio of about 1:7 and a unilateral incidence of about 90%, with left side more likely to be affected than right side (3:1) [30]. In our group, there were 9 boys and 30 girls, with the incidence of DDH in girls much higher than that in boys. This may have something to do with genetics [30]. In addition, during childbirth, the mother will secrete a large amount of relaxation hormone, in order to achieve the purpose of relaxing ligaments and expanding the birth canal. Girls are more sensitive to this relaxation hormone than boys, and have more loose soft tissue around the hip. If there is resistance during delivery, hip dislocation is more likely to occur, which is also the reason why DDH rates are higher in girls than boys.

DDH is more common in the left hip [30]. In this group of DDH cases, there were 25 left hip lesions and 18 right hip lesions. Due to the insufficient sample size, the difference of prediction accuracy of left and right hip joints was not further studied.

This study has some limitations. It was a retrospective study, and the sample size of this study was small. Given these limitations, prospective and global sample studies may be necessary to confirm the diagnostic capability of MRI.

## Conclusions

In conclusion, MR could adequately detect thickened fibrofatty pulvinar tissue, joint effusion, inversion of the labrum, and iliopsoas muscle atrophy, thickened round ligament. These irreducible factors can be observed by MRI before surgery and help orthopedist select a suitable operation such as closed or open reduction. MRI technology can provide abundant morphological information for acetabular development and has important clinical value for DDH diagnosis and surgical treatment.

## Data Availability

Most of the data supporting our findings is contained within the manuscript, and all others, excluding identifying/confidential respondent data, will be shared upon request.

## Abbreviations

DDH: The developmental dysplasia of the hip
MRI: Magnetic resonance imaging
ACR: American College of Radiology
US: Ultrasonography
DR: Digital radiography
AVN: Avascular necrosis
T1-W: T1-weighted
SE: Spin-echo
TR: Repetition time
TE: Echo time
STIR: Short inversion time inversion recovery
PACS: The Picture Archiving and Communication Systems
PPV: Positive predictive values
NPV: Negative predictive values
ICC: Intra-class correlation coefficient
CI: Confidence interval
